# Could Metabolic Syndrome Be a Predictor of Survival Outcomes in Upper Tract Urothelial Carcinoma? A Propensity Score Matching Study in a Large Chinese Center

**DOI:** 10.3389/fonc.2022.816915

**Published:** 2022-05-26

**Authors:** Xiang Dai, Fei Wang, Yiqing Du, Caipeng Qin, Shicong Lai, Yuxuan Song, Zixiong Huang, Songchen Han, Xiaopeng Zhang, Tao Xu

**Affiliations:** Department of Urology, Peking University People’s Hospital, Beijing, China

**Keywords:** metabolic syndrome, upper tract urothelial carcinoma, propensity score matching, prognosis, survival

## Abstract

**Purpose:**

To evaluate the prognostic value of metabolic syndrome (MetS) in upper tract urothelial carcinoma (UTUC) patients based on propensity score matching (PSM) analysis.

**Patients and Methods:**

A total of 573 patients with UTUC after radical nephroureterectomy were included at Peking University People’s Hospital from January 2007 to April 2021. MetS was diagnosed according to the criteria of Chinese Diabetes Society and was defined as the presence of 3 or more of the following 4 conditions (obesity, hyperglycemia, hypertension, high triglycerides and/or low high-density lipoprotein-cholesterol). Patients were divided into two groups based on whether they had MetS, whose variables were adjusted using 1:1 PSM analysis with a caliber of 0.02 to minimize selection bias. Univariate and multivariate Cox regression analysis were used to evaluate the association of MetS and its components with pathological outcomes after adjusting preoperative confounders by propensity score matching. The Kaplan-Meier method was used to estimate overall survival (OS), cancer-specific survival (CSS), and intravesical recurrence-free survival (IVRFS) after surgery.

**Results:**

MetS was significantly correlated with older age, a history of coronary heart disease, high Charlson Comorbidity Index, low estimated Glomerular filtration rate, and low aspartate/alanine aminotransferase ratio (all P<0.05). Multivariate Cox regression analysis and Kaplan-Meier curves demonstrated that MetS showed no statistical correlation with lower OS or IVRFS and approaching significance with lower CSS (P=0.063) before PSM. After PSM, the 5-year OS, CSS, and IVRFS were 64.1%, 74.7%, and 77.2%, respectively, in the MetS group, compared with 67.4%, 78.8%, and 77.2%, respectively, in non-MetS group. Univariate Cox regression analyses showed that MetS and its components were not associated with decreased OS, CSS, or IVRFS (all P>0.05).

**Conclusion:**

In our study, no statistical difference was found between MetS and survival outcomes in UTUC, except a marginal association with lower CSS. Further studies are needed to evaluate the role of MetS and its each single component on UTUC.

## Introduction

There is a high incidence of urothelial carcinoma and it ranks among the top ten malignant tumors worldwide. Bladder urothelial carcinoma accounts for its vast majority. Upper tract urothelial carcinoma (UTUC) accounts for only 5-10% of the total urothelial carcinoma but the proportion is higher in the Asian population, at about 9.3-29.9%, with an average of 17.9% (1). Therefore, UTUC may have different pathogenesis and clinical characteristics in the Asian population.

Metabolic Syndrome (MetS) is defined as a group of clinical manifestations including obesity, hyperglycemia, dyslipidemia (hypertriglyceridemia and/or hypo-high-density-lipoproteinemia), and hypertension. The components mentioned above seriously affected the health and showed aggregation at the onset. There is now increasing evidence of an association between MetS and tumor development and prognosis, such as colorectal cancer (2), breast cancer (3) and urinary cancer including prostate cancer (4) and bladder cancer (5). Few studies have investigated the link between MetS and UTUC, including one cohort study and two studies based on Surveillance, Epidemiology and End Results (SEER)-Medicare linked database (6–8). Although their studies showed that the patients with MetS had inferior survival outcomes, they may not be able to select the most suitable diagnostic criteria of MetS for the Chinese population. In 2004, the Chinese Diabetes Society (CDS) released MetS’s diagnostic criteria for the Chinese population (9), and reaffirmed the criteria in the latest consensus in 2019. In the determination of obesity, CDS adopts the same index as the American Heart Association/National Heart, Lung, and Blood Institute (AHA/NHLBI) but with a lower cut-off (BMI≥25kg/m^2^ vs BMI≥28kg/m^2^). This is different from International Diabetes Federation (IDF), which selects waist circumference as the evaluation criteria of centripetal obesity. At the same time, the CDS combined hypertriglyceridemia and hypo-high-density-lipoproteinemia. In general, the CDS takes into account the baseline characteristics of the lower BMI in the Asian population and imposes stricter requirements on the diversity of metabolic abnormalities. The aim of our study was to evaluate the prognostic value of MetS in a large Chinese cohort based on the diagnostic criteria from CDS using propensity score matching (PSM) analysis.

## Material and Methods

### Study Population

**Figure 1:**
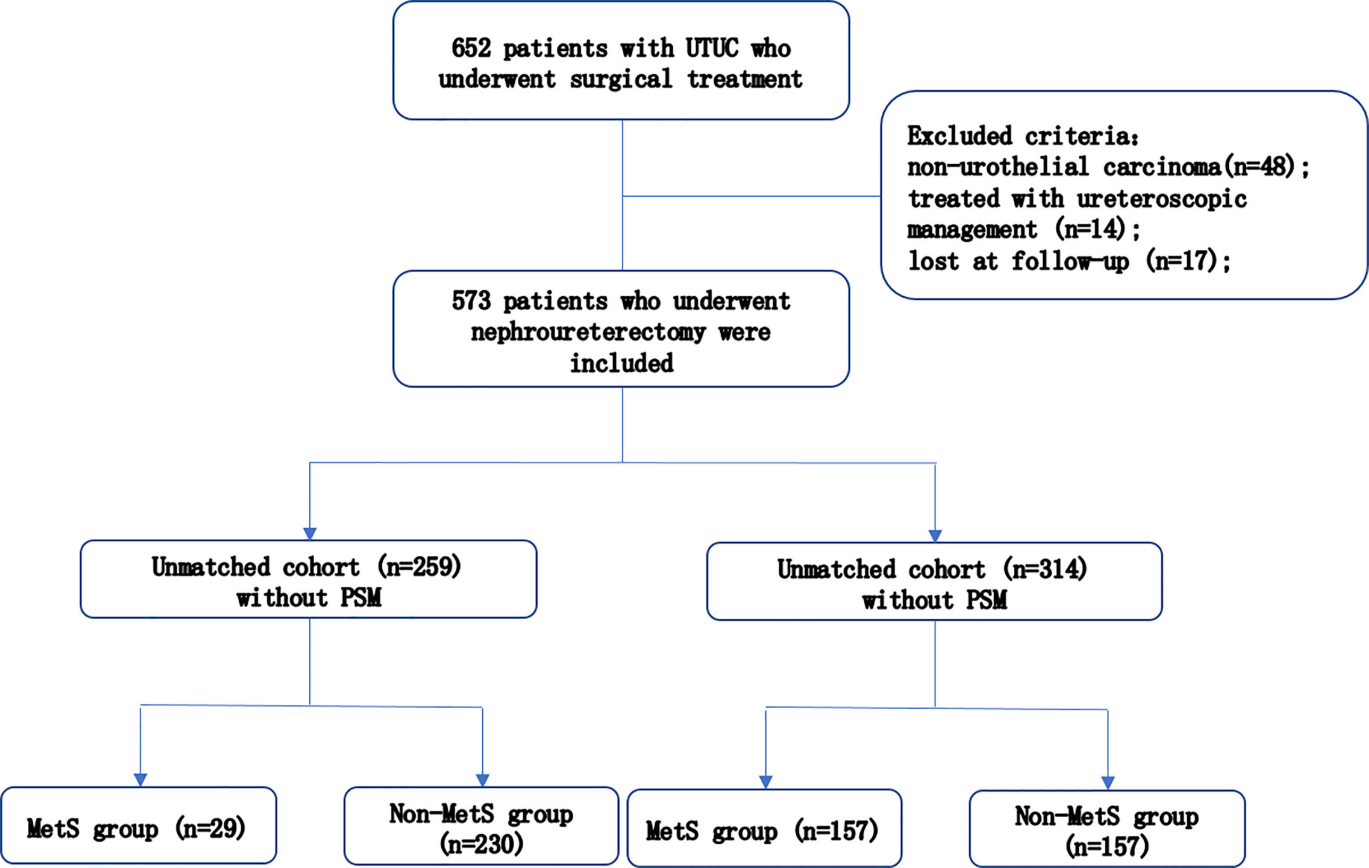
Patient selection flowchart.

This study received the approval from the Internal Ethics Review Board of the Peking University People’s Hospital. We retrospectively collected the clinical and pathological records of 652 patients diagnosed with UTUC. We excluded 79 patients because they had non-urothelial carcinoma (n=48); were treated with ureteroscopic management (n=14); or did not show at the follow-up appointment (n=17). In total, 573 patients were included for further study (**Figure 1**). They were treated with radical nephroureterectomy (RNU) from January 2007 to April 2021. The type of bladder cuff removal included transvesical, extravesical, and endoscopic.

Lymph node dissection (LND) was performed when invasive UTUC was suspected or suspicious lymph node metastasis was found by preoperative imaging. The pathological staging was assessed according to the 2002 Union for International Cancer Control (UICC) Tumor-node-metastasis (TNM) classification system. Tumor grade was determined according to the World Health Organization/International Society of Urologic Pathology 2004 classification (WHO/ISUP) grading system. Other pathological features were simultaneously retrieved from the pathological reports.

### MetS Definitions

MetS was diagnosed according to the criteria of CDS and was defined as the presence of 3 or more of the following 4 conditions: (1) obesity: body mass index (BMI)≥25kg/m^2^; (2) hyperglycemia: fasting plasma glucose (FPG) ≥ 6.1mmol/L and/or 2-hour postprandial blood glucose (2hPG) ≥ 7.8mmol/L or drug treatment for any type of diabetes mellitus, (3) high blood pressure: systolic blood pressure≥140 mmHg or diastolic blood pressure≥90 mmHg or antihypertensive drug treatment, or (4) high triglycerides (defined as ≥1.7mmol/L) and/or low high-density lipoprotein-cholesterol (defined as <0.9mmol/L in males and <1.0mmol/L in females).

### Follow-Up

Before the operation, blood and urinary samples were routinely obtained. For patients who were followed, cystoscopy was done every 3 months for the first 2 years after RNU and once a year thereafter. Computed tomography or magnetic resonance imaging, blood and urinary laboratory tests, and other evaluations were also performed. Overall survival (OS) was evaluated from the date of surgery to the date of death from all causes. Cancer-specific survival (CSS) was defined as the interval between surgery and cancer-specific death. Intravesical recurrence-free survival (IVRFS) was defined as the interval between surgery and identification of a subsequent bladder tumor during cystoscopy confirmed by pathological evaluation.

### Statistical Analysis

Differences in clinical and pathological characteristics among MetS and non-MetS groups were compared using Chi-square test for categorical variables and Student’s t-test or Mann-Whitney U test for continuous variables. Continuous variables with normal distribution were present as mean ± standard deviation (SD) and non-normal variables were reported as median (interquartile range). Univariate and multivariate Cox regression analysis were used to evaluate the association of MetS and its components with pathological outcomes after adjusting preoperative confounders by propensity score matching. All factors with p-value<0.1 in univariate Cox regression analysis were included in multivariate analysis. Patients were divided into two groups based on whether they had MetS, or whether variables were adjusted using 1:1 PSM analysis with a caliber of 0.02 to minimize selection bias. Standardized differences were used to compared the balance of baseline characteristics and covariates between MetS and non-MetS patients before and after matching, expressed as absolute standardized difference. The Kaplan-Meier method was used to estimate OS, CSS, and IVRFS after surgery. Statistical analyses were performed using IBM SPSS Statistics version 24.0 (IBM Corp., Armonk, NY, USA) and Stata version 15 (StataCorp LLC, Texas, USA). A two-sided P value <0.05 was considered statistically significant.

## Results

### Clinicopathological Characteristics Before PSM

Of the 573 UTUC patients in the entire cohort, 186 (32.5%) were diagnosed with MetS. The baseline characteristics of patients before PSM are demonstrated in **Table 1**. Median age of patients at surgery was 67.27 ± 9.97 year, with a median follow-up duration of 32.8 months (range from 1 to 179 months). Low-grade and high-grade UTUC were seen in 106 patients (18.5%) and 456 patients (79.6%), respectively, and 11 patients (1.9%) were in unclear classification. Non-muscle-invasive UTUC (pTa-Tis-T1) was seen in 145 (25.3%) of patients, 125 (21.8%) showed pT2, 274 (47.8%) showed pT3 or pT4, and 29 (5.1%) showed unclear results. A total of 230 patients (40.1%) were diagnosed with obesity, 176 patients (30.7%) with hyperglycemia, 371 patients (64.7%) with hypertension, and 313 patients (54.6%) with hyperlipidemia. Patients with MetS were significantly older (P=0.04), more likely to have a history of coronary heart disease (CHD) (P=0.002); had a higher Charlson Comorbidity Index (CCI) (P<0.001), a lower estimated Glomerular filtration rate(eGFR) (P=0.014), and lower AST/ALT ratio (P<0.001). Each component of MetS was also significantly higher in the MetS group, compared with patients in the non-MetS group. Although differences of these baseline characteristics and laboratory tests were statistically significant, there was no statistical difference in pathological characteristics including tumor stage and grade.

**Table 1:** Clinicopathological characteristics of the entire cohort and subgroups according to MetS before propensity score matching.

Characteristics	Subgroup	Entire cohort	MetS	Non-MetS	P-value
Number		573	186	387	
Preoperative characteristics					
Age, years		67.27 ± 9.97	68.51 ± 9.41	66.68 ± 10.19	0.040
Gender	Male	292 (51.0%)	91 (48.9%)	201 (51.9%)	0.500
	Female	281 (49.0%)	95 (51.1%)	186 (48.1%)	
Tobacco	Yes	98 (17.1%)	34 (18.3%)	64 (16.5%)	0.605
	No	475 (82.9%)	152 (81.7%)	323 (83.5%)	
CHD	Yes	77 (13.4%)	37 (19.9%)	40 (10.3%)	0.002
	No	496 (86.6%)	149 (80.1%)	347 (89.7%)	
CCI		3.15 ± 1.55	3.54 ± 1.67	2.96 ± 1.46	<0.001
ASA	1	42 (7.3%)	34 (8.8%)	8 (4.3%)	0.069
	2	436 (76.1%)	293 (75.7%)	143 (76.9%)	
	3	95 (16.6%)	60 (15.5%)	35 (18.8%)	
PLR		153.13 ± 74.16	144.99 ± 65.16	157.04 ± 77.89	0.068
Hb, g/dl		125.4 ± 18.09	124.12 ± 19.14	126.01 ± 17.56	0.242
eGFR, ml/min/1.73m^2^		67.28 ± 23.51	63.80 ± 25.12	68.95 ± 22.55	0.014
FAR		8.64 ± 2.57	8.74 ± 2.70	8.59 ± 2.50	0.497
AAR		1.36 ± 0.59	1.21 ± 0.43	1.43 ± 0.63	<0.001
TG, mmol/L		1.58 ± 1.14	2.00 ± 1.66	1.38 ± 0.70	<0.001
HDL-C, mmol/L		1.11 ± 0.26	1.00 ± 0.24	1.17 ± 0.25	<0.001
GLU, mmol/L		5.89 ± 1.83	6.96 ± 2.44	5.37 ± 1.13	<0.001
Components of MetS					
BMI	≥25	230 (40.1%)	152 (81.7%)	78 (20.1%)	<0.001
	<25	343 (59.9%)	34 (18.3%)	309 (79.8%)	
Diabetes	Yes	176 (30.7%)	122 (65.6%)	54 (14.0%)	<0.001
	No	397 (69.3%)	64 (34.4%)	333 (86.0%)	
Hypertension	Yes	371 (64.8%)	172 (92.5%)	199 (51.4%)	<0.001
	No	202 (35.3%)	14 (7.5%)	188 (48.6%)	
Hyperlipidemia	Yes	313 (54.6%)	167 (89.8%)	146 (37.7%)	<0.001
	No	260 (45.4%)	19 (10.2%)	241 (62.3%)	
Pathological characteristics					
T stage	T_a_-T_1_	145 (25.3%)	49 (26.3%)	96 (24.8%)	0.252
	T_2_	125 (21.8%)	45 (24.2%)	80 (20.7%)	
	T_3-4_	274 (47.8%)	80 (43.0%)	194 (50.1%)	
	Undefined	29 (5.1%)	13 (7.0%)	16 (4.1%)	
N stage	N_0_	71 (12.4%)	20 (10.8%)	51 (13.2%)	0.360
	N_1_	18 (3.1%)	3 (1.6%)	15 (3.9%)	
	N_x_	484 (84.5%)	163 (87.6%)	321 (82.9%)	
WHO/ISUP grade	Low Grade	106 (18.5%)	37 (19.9%)	69 (17.8%)	0.599
	High Grade	456 (79.6%)	147 (79.0%)	309 (79.8%)	
	Undefined	11 (1.9%)	3 (1.6%)	8 (2.1%)	
Tumor diameter, cm		3.21 ± 1.98	3.09 ± 1.84	3.27 ± 2.04	0.306
Multifocality	Single	447 (78.0%)	151 (81.2%)	296 (76.5%)	0.204
	Multi	126 (22.0%)	35 (18.8%)	91 (23.5%)	
CIS	Yes	28 (4.9%)	5 (2.7%)	23 (6.0%)	0.091
	No	545 (95.1%)	181 (97.3%)	364 (94.1%)	

CHD, coronary heart disease; CCI,Charlson Comorbidity Index; ASA, American Society of Anesthesiologists classification; PLR, platelet-lymphocyte ratio; Hb, hemoglobin; eGFR, estimated Glomerular filtration rate; FAR, fibrinogen-albumin ratio; AAR, aspartate/alanine aminotransferase ratio; TG, triglyceride; HDL-C, high density liptein cholesterol; Glu, glucose; BMI, body mass index; CIS, carcinoma in situ.

### Survival Outcomes Before PSM

**Figure 2:**
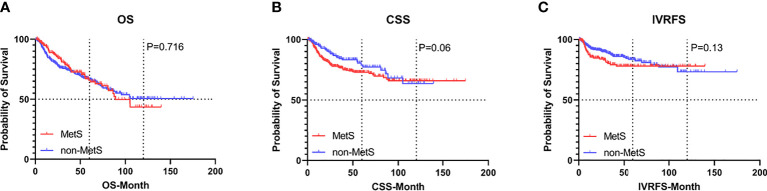
Kaplan-Meier curves for survival outcomes in UTUC patients according to the presence of MetS before propensity score matching. **(A)** OS, **(B)** CSS, and **(C)** IVRFS. OS, overall survival; CSS, cancer-specific survival; IVRFS, intravesical recurrence-free survival.

Before PSM, with a median follow-up of 32.8 months, there were 169 (29.5%) overall deaths after a median (interquartile range [IQR]) of 20 (9.8-40.0) months and 121 (21.1%) cancer-specific deaths after a median (IQR) of 15.1 (8.4-27.5) months, postoperatively. Also, 79 patients (13.8%) experienced intravesical recurrence (IVR) after a median (IQR) of 10.5 (6.4-28.5) months. After controlling for clinicopathological characteristics, there was a borderline effect in which members of the MetS group demonstrated a somewhat lower CSS compared with patients without MetS (95% confidence interval [CI] 0.978-2.351, P=0.063) based on multivariate Cox regression analyses, as shown in **Table 2**. Moreover, patient gender, pathologic T stage, tumor grade, and tumor size were revealed as significant co-predictors of CSS. However, MetS was not found to be an independent predictor for OS (95%CI 0.683-1.300, p=0.716) and IVRFS (95%CI 0.590-1.122, p=0.361). For OS, patient gender (P=0.029), pathologic T stage (P=0.010), tumor grade (P=0.002), and tumor size (P=0.001) were also significant co-predictors. Patient gender (P=0.009), PLR (P=0.001) and tumor multifocality (P=0.002) were revealed as independent predictors for IVRFS. Kaplan-Meier curves demonstrated that MetS showed no statistical correlation with lower OS and IVRFS and a marginal association with lower CSS (P=0.06) than those without MetS (**Figures 2A–C**).

**Table 2:** Univariate and multivariate cox regression analyses for OS, CSS and IVRFS before propensity score matching.

Characteristics	OS				CSS				IVRFS			
	Univariate		Multivariate		Univariate		Multivariate		Univariate		Multivariate	
	P	95%CI	P	95%CI	P	95%CI	P	95%CI	P	95%CI	P	95%CI
Age, years	**0.001**	**1.012-1.045**	0.154	0.994-1.039	0.076	0.998-1.036	0.483	0.984-1.035	**0.026**	**1.003-1.051**	0.264	0.987-1.050
Gender	**0.031**	**0.528-0.970**	**0.029**	**0.462-0.959**	**0.037**	**0.475-0.977**	**0.029**	**0.408-0.953**	**0.018**	**0.368-0.911**	**0.009**	**0.339-0.856**
Tobacco	0.611	0.611-1.335			0.224	0.444-1.210			0.365	0.406-1.392		
coronary	0.117	0.919-2.129			0.924	0.597-1.766			0.425	0.694-2.380		
CCI	**0.001**	**1.110-1.348**	0.488	0.906-1.231	**0.032**	**1.011-1.272**	0.467	0.893-1.280	0.053	0.998-1.327	0.422	0.886-1.336
ASA												
1	–	Referent			–	Referent			–	Referent		
2	**0.006**	**0.141-0.726**	0.203	0.238-1.356	0.169	0.207-1.318			0.734	0.316-2.252		
3	**0.023**	**0.431-0.941**	0.868	0.602-1.535	0.712	0.549-1.507			0.760	0.487-1.691		
PLR	**0.002**	**1.001-1.004**	0.381	0.999-1.003	**0.002**	**1.001-1.005**	0.669	0.998-1.003	**0.001**	**1.002-1.006**	**0.001**	**1.002-1.006**
Hb, g/dl	**0.001**	**0.979-0.995**	0.278	0.983-1.005	**0.004**	**0.977-0.995**	0.103	0.977-1.002	0.778	0.989-1.014		
eGFR, ml/min/1.73m^2^	**0.001**	**0.983-0.995**	0.612	0.990-1.006	0.061	0.986-1.000	0.657	0.993-1.011	0.248	0.986-1.004		
AAR	0.458	0.876-1.342			0.149	0.943-1.473			0.879	0.677-1.397		
FAR	**0.001**	**1.058-1.152**	0.443	0.967-1.081	**0.001**	**1.059-1.171**	0.306	0.968-1.109	0.139	0.454-1.116		
MetS	0.716	0.683-1.300			0.094	0.943-2.117	0.063	0.978-2.351	0.088	0.990-1.153	0.361	0.59-1.122
BMI (>25kg/m^2^)	0.421	0.646-1.200			0.716	0.491-1.043			0.596	0.723-1.758		
Diabetes	0.143	0.924-1.731			0.998	0.680-1.473			0.779	0.666-1.720		
Hypertension	0.673	0.779-1.473			0.654	0.636-1.329			0.726	0.680-1.739		
Hyperlipidemia	0.481	0.663-1.214			0.294	0.578-1.181			0.901	0.624-1.514		
T (≥2) stage	**0.001**	**2.498-7.025**	**0.010**	**0.257-0.828**	**0.001**	**3.600-18.630**	**0.006**	**0.127-0.705**	0.123	0.894-2.565		
WHO grade	**0.001**	**2.374-7.403**	**0.002**	**0.202-0.694**	**0.001**	**2.687-13.931**	**0.003**	**0.100-0.634**	0.437	0.710-2.210		
Tumor diameter(>=3cm)	**0.001**	**1.265-2.389**	**0.001**	**1.113-1.313**	**0.011**	**1.115-2.357**	**0.001**	**1.095-1.315**	0.291	0.814-1.986		
Multifocality	0.704	0.635-1.359			0.662	0.717-1.688			**0.005**	**1.231-3.149**	**0.002**	**0.294-0.761**
CIS	0.426	0.318-1.623			0.995	0.439-2.267			0.584	0.228-2.298		

CCI, Charlson Comorbidity Index; ASA. American Society of Anesthesiologists classification; PLR, platelet-lymphocyte ratio; Hb, hemoglobin; eGFR, estimated Glomerular filtration rate; FAR, fibrinogen-albumin ratio; BMI, body mass index; CIS, carcinoma in situ.

Bold values represent statistical differences.

### Clinicopathological Characteristics After PSM

**Figure 3:**
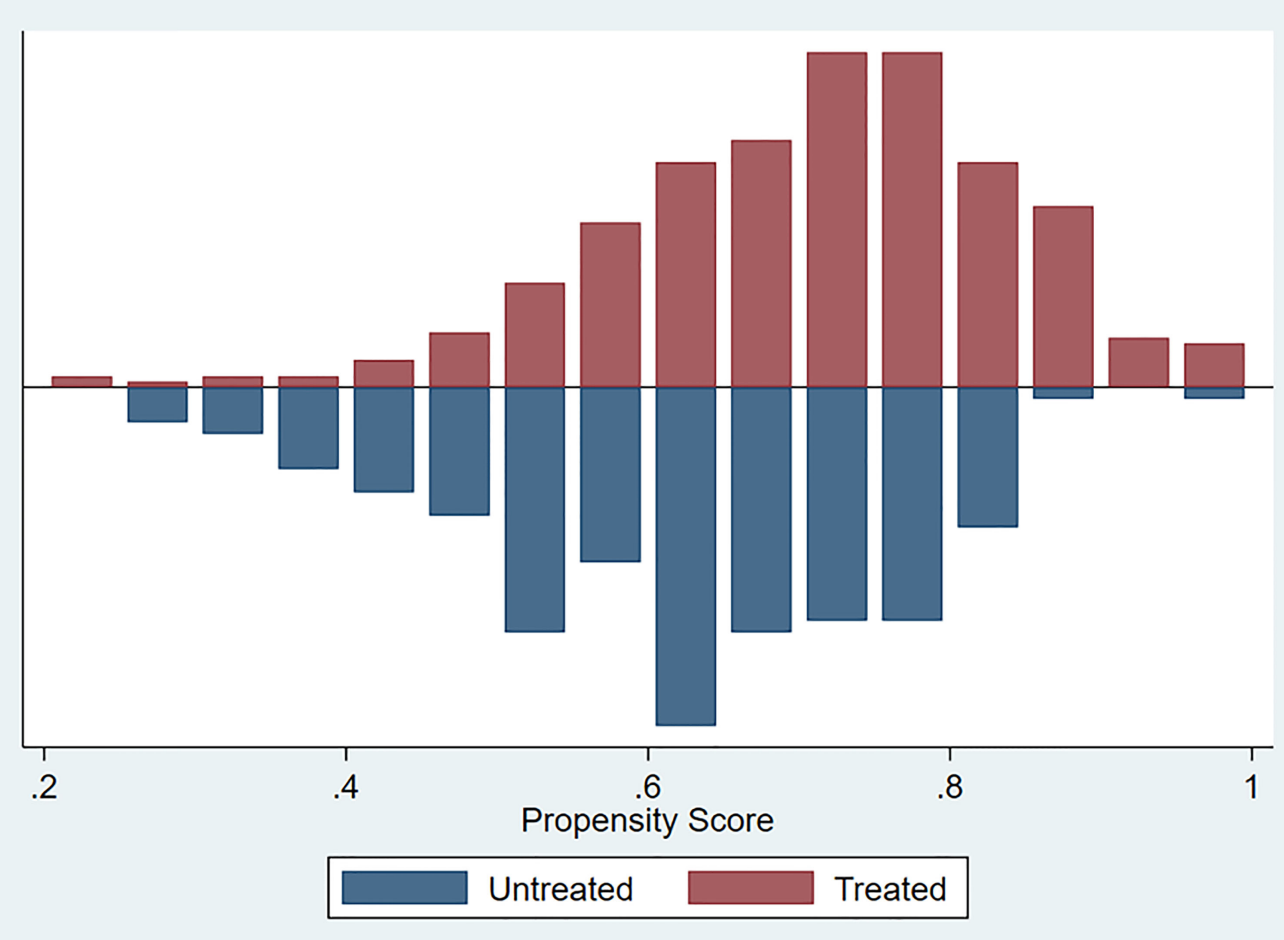
Covariate balance test of matching.

**Figure 4:**
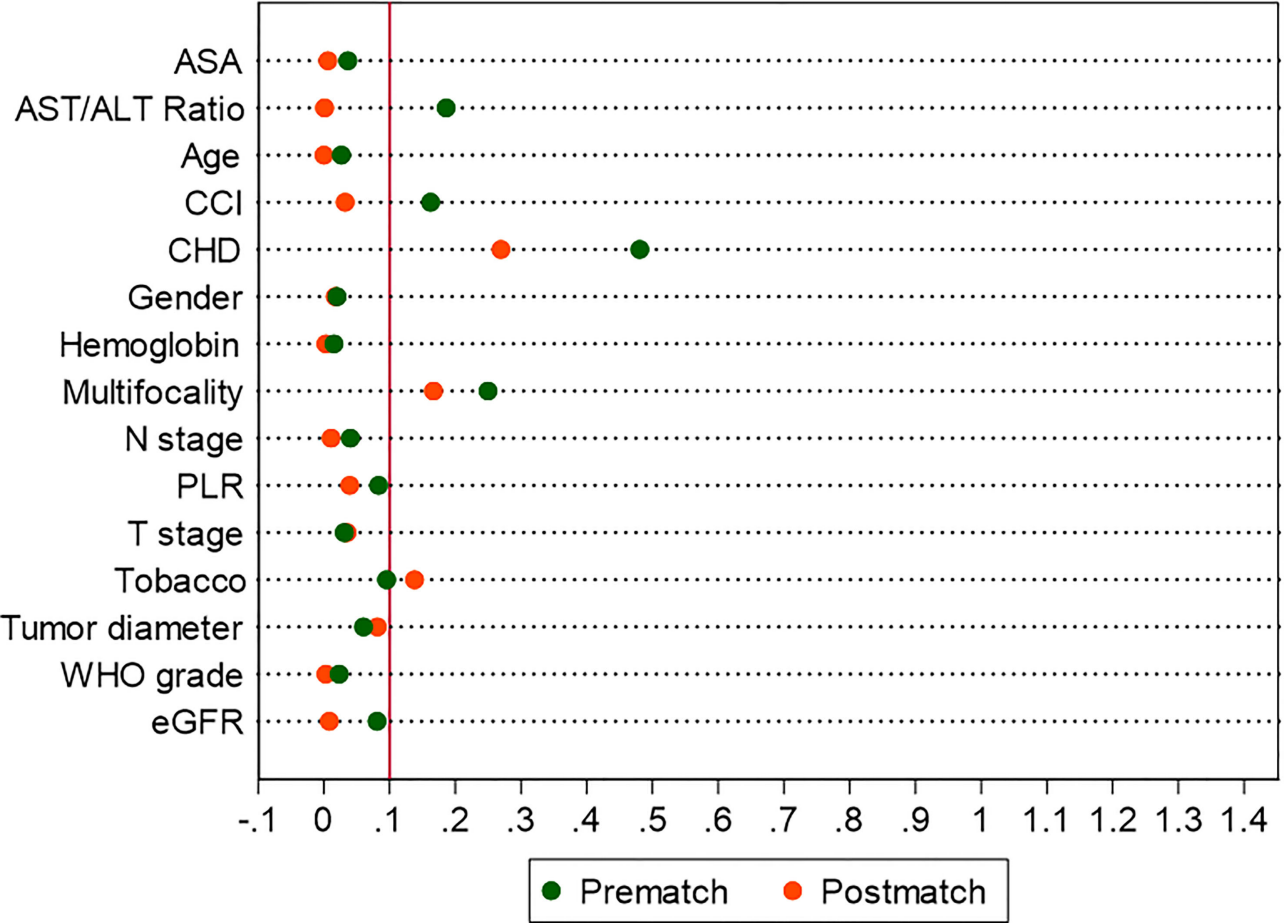
Absolute standardized differences in all characteristics between MetS and non-MetS group, before and after propensity score matching.

**Figure 5:**
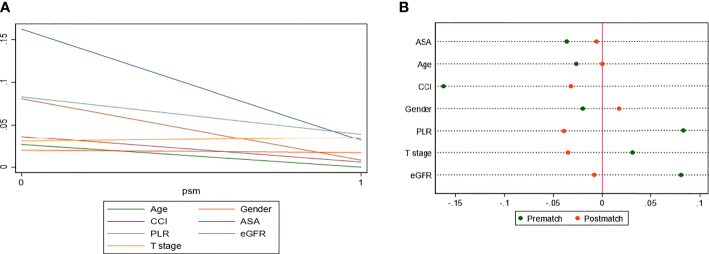
Standardized differences in covariates between MetS and non-MetS group, prematch and postmatch: **(A)** Line-plot; **(B)** Dot-plot.

After PSM, the distributions of baseline and clinicopathological characteristics between MetS and non-MetS groups are summarized **Table 3**. Absolute standardized differences for all observed covariates were below 15%, suggesting an acceptable improvement in covariate balance. Only the standard difference of CHD, which is closely associated with MetS, declined less than other covariates. Age, gender, CCI, ASA classification, PLR, eGFR, AST/ALT ratio, and T stage were controlled in matching with a caliber of 0.02 (**Figures 3**–**5**). As shown in **Table 3**, nearly all clinicopathological characteristics did not have any statistical difference between MetS and non-MetS group, except each component of MetS diagnosis (all P<0.05).

**Table 3:** Clinicopathological characteristics of the entire cohort and subgroups according to MetS after propensity score matching.

Characteristics	Subgroup	Entire cohort	MetS	Non-MetS	P-value
N		314	157	157	
Preoperative characteristics					
Age, years		67.92 ± 9.60	67.92 ± 9.43	67.92 ± 9.80	1.000
Gender	Male	156 (49.7%)	80 (51.0%)	76 (48.4%)	0.653
	Female	158 (50.3%)	77 (49.0%)	81 (51.6%)	
Tobacco	Yes	54 (17.2%)	29 (18.5%)	25 (15.9%)	0.551
	No	260 (82.8%)	128 (81.5%)	132 (84.1%)	
CHD	Yes	45 (14.3%)	26 (16.6%)	19 (12.1%)	0.260
	No	269 (85.7%)	131 (83.4%)	138 (87.9%)	
CCI		3.32 ± 1.53	3.37 ± 1.62	3.26 ± 1.43	0.531
ASA	1	15 (4.8%)	7 (4.5%)	8 (5.1%)	0.800
	2	248 (79.0%)	124 (79.0%)	124 (79.0%)	
	3	51 (16.2%)	26 (16.6%)	25 (15.9%)	
PLR		146.35 ± 60.43	149.28 ± 67.4	143.42 ± 52.61	0.391
Hb, g/dl		124.63 ± 18.17	124.44 ± 19.04	124.81 ± 17.32	0.859
eGFR, ml/min/1.73m^2^		65.76 ± 23.37	66.03 ± 24.21	65.48 ± 22.57	0.837
FAR		8.64 ± 2.40	8.61 ± 2.38	8.67 ± 2.43	0.849
AAR		1.24 ± 0.42	1.24 ± 0.44	1.24 ± 0.41	0.980
TG, mmol/L		1.69 ± 1.38	1.97 ± 1.77	1.41 ± 0.68	<0.001
HDL-C, mmol/L		1.07 ± 0.25	1.01 ± 0.25	1.13 ± 0.24	<0.001
GLU, mmol/L		6.12 ± 2.04	6.85 ± 2.48	5.40 ± 1.08	<0.001
Components of MetS					
BMI		24.91 ± 3.46	26.62 ± 2.97	23.21 ± 3.05	<0.001
Diabetes	Yes	125 (39.8%)	100 (63.7%)	25 (15.9%)	<0.001
	No	189 (60.2%)	57 (36.3%)	132 (84.1%)	
Hypertension	Yes	232 (73.9%)	146 (93.0%)	86 (54.8%)	<0.001
	No	82 (26.1%)	11 (7.0%)	71 (45.2%)	
Hyperlipidemia	Yes	202 (64.3%)	141 (89.8%)	61 (38.9%)	<0.001
	No	112 (35.7%)	16 (10.2%)	96 (61.1%)	
T stage	T_a_-T_1_	90 (28.7%)	43 (27.4%)	47 (29.9%)	0.948
	T_2_	71 (22.6%)	40 (15.5%)	31 (19.7%)	
	T_3-4_	153 (48.7%)	74 (47.1%)	79 (50.3%)	
N stage	N_0_	40 (12.7%)	20 (12.7%)	20 (12.7%)	0.377
	N_1_	7 (2.2%)	2 (1.3%)	5 (3.2%)	
	N_x_	267 (85.0%)	135 (86.0%)	132 (84.1%)	
WHO/ISUP	LG	58 (18.5%)	29 (18.5%)	29 (18.5%)	0.957
	HG	252 (80.3%)	127 (80.9%)	125 (79.6%)	
	Undefined	4 (1.3%)	1 (0.6%)	3 (1.9%)	
Tumor diameter		3.06 ± 1.75	3.19 ± 1.90	2.93 ± 1.57	0.474
Multifocality	Single	249 (79.3%)	127 (80.9%)	122 (77.7%)	0.488
	Multi	65 (20.7%)	30 (19.1%)	35 (22.3%)	
CIS	Yes	17 (5.4%)	5 (3.2%)	12 (7.6%)	0.081
	No	297 (94.6%)	152 (96.8%)	145 (92.4%)	

CHD, coronary heart disease; CCI, Charlson Comorbidity Index; ASA, American Society of Anesthesiologists classification; PLR, platelet-lymphocyte ratio; Hb, hemoglobin; eGFR, estimated Glomerular filtration rate; FAR, fibrinogen-albumin ratio; AAR, aspartate/alanine aminotransferase ratio; TG, triglyceride; HDL-C, high density liptein cholesterol; Glu, glucose; BMI, body mass index; CIS, carcinoma in situ.

### Survival Outcomes After PSM

**Figure 6:**
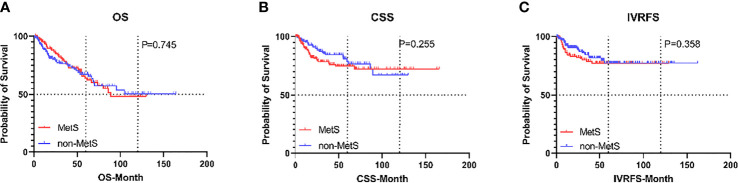
Kaplan-Meier curves for survival outcomes in UTUC patients according to the presence of MetS after propensity score matching. **(A)** OS, **(B)** CSS, and **(C)** IVRFS. OS, overall survival; CSS, cancer-specific survival; IVRFS, intravesical recurrence-free survival.

With a median (IQR) follow-up duration of 33.1 (14.0-58.6) months postoperatively, there were 90 (28.7%) overall deaths after a median (IQR) of 32.6 (13.9-68.1) months and 58 (18.5%) cancer-specific deaths after a median (IQR) of 17.7 (9.7-39.1) months. Also, 50 patients (15.9%) experienced IVR after a median (IQR) of 10.1 (6.4-23.5) months. The 5-year OS, CSS, and IVRFS were 64.1%, 74.7%, and 77.2%, respectively, in the MetS group, as compared with 67.4%, 78.8%, and 77.2%, respectively, in non-MetS group. Kaplan-Meier curves demonstrated that MetS patients had almost the same CSS, RFS, and OS as those without MetS (all P>0.05; **Figure 6**). Univariate Cox proportional hazards regression analyses showed that MetS and its components were not associated with decreased OS, CSS, and IVRFS (all P>0.05; **Table 4**). After adjusting clinical confounders, multivariate Cox regression analysis showed that age, pathological T stage, tumor grade, and tumor size were significant co-predictors of OS (all P<0.05). T stage and tumor grade were also significant co-predictors of CSS (both P<0.05). Age and patient gender were significant co-predictors of IVRFS (both P<0.05) (**Table 4**).

**Table 4:** Univariate and multivariate cox regression analyses for OS, CSS and IVRFS after propensity score matching.

Characteristics	OS				CSS				IVRFS			
	Univariate		Multivariate		Univariate		Multivariate		Univariate		Multivariate	
	P	95%CI	P	95%CI	P	95%CI	P	95%CI	P	95%CI	P	95%CI
Age, years	**0.005**	**1.010-1.058**	**0.026**	**1.003-1.057**	0.159	0.992-1.049			**0.014**	**1.008-1.073**	**0.010**	**1.010-1.075**
Gender	0.056	0.438-1.011	0.133	0.404-1.127	0.110	0.387-1.101			0.062	0.972-3.019	**0.047**	**0.315-0.980**
Tobacco	0.949	0.586-1.651			0.481	0.634-2.633			0.395	0.636-3.145		
coronary	0.440	0.689-2.354			0.664	0.557-2.501			0.487	0.613-2.792		
CCI	0.615	0.414-1.684			0.820	0.427-2.933			0.875	0.348-2.454		
ASA												
1	–	Referent			–	Referent			–	Referent		
2	0.302	0.102-2.030			0.568	0.062-4.584			0.605	0.420-4.437		
3	0.797	0.500-1.702			0.267	0.671-4.225			0.332	0.335-1.446		
PLR	0.735	0.996-1.003			0.993	0.996-1.004			0.736	0.997-1.005		
Hb, g/dl	0.069	0.979-1.001	0.394	0.979-1.009	**0.032**	**0.972-0.999**	0.119	0.974-1.003	0.406	0.991-1.023		
eGFR, ml/min/1.73m^2^	0.062	0.984-1.000	0.915	0.991-1.011	0.358	0.985-1.006			0.310	0.983-1.006		
AAR	**0.004**	**1.035-1.196**	0.785	0.917-1.121	0.065	0.994-1.200			0.583	0.924-1.150		
FAR	0.172	0.878-2.081			0.069	0.964-2.637			0.562	0.650-2.211		
MetS	0.745	0.617-1.412			0.257	0.440-1.245			0.360	0.743-2.269		
BMI (>25kg/m^2^)	0.953	0.670-1.531			0.628	0.678-1.904			0.452	0.463-1.409		
Diabetes	0.334	0.537-1.235			0.871	0.615-1.777			0.442	0.700-2.259		
Hypertension	0.392	0.771-1.942			0.155	0.860-2.580			0.356	0.726-2.441		
Hyperlipidemia	0.330	0.809-1.881			0.303	0.780-2.226			0.901	0.582-1.849		
T (≥2) stage	**0.001**	**0.132-0.491**	**0.037**	**0.209-0.952**	**0.001**	**0.036-0.369**	**0.033**	**0.080-0.902**	0.213	0.346-1.268		
WHO grade	**0.001**	**0.121-0.569**	**0.048**	**0.189-0.993**	**0.004**	**0.030-0.501**	**0.072**	**0.062-1.125**	0.186	0.261-1.298		
Tumor diameter(>=3cm)	**0.003**	**0.329-0.790**	**0.001**	**1.090-1.387**	**0.024**	**0.308-0.922**	**0.004**	**1.065-1.392**	0.117	0.366-1.118		
Multifocality	0.369	0.740-2.246			0.850	0.552-2.055			0.248	0.366-1.296		
CIS	0.488	0.475-4.760			0.990	0.310-3.175			0.613	0.350-5.928		

CCI, Charlson Comorbidity Index; ASA, American Society of Anesthesiologists classification; PLR, platelet-lymphocyte ratio; Hb, hemoglobin; eGFR, estimated Glomerular filtration rate; FAR, fibrinogen-albumin ratio; BMI, body mass index; CIS, carcinoma in situ. Bold values represent statistical differences.

## Discussion

In our present single-center study, 573 patients with UTUC treated with RNU were included and we observed whether MetS had negative impact on survival outcomes. Moreover, we re-evaluated the association between MetS and outcomes of UTUC after adjusting preoperative confounders by propensity score matching with a sample size of 314 patients. The results showed that the existence of MetS was not an independent factor for worse pathological outcomes and survival outcomes including death or IVR. However, it is worth noting that there presented a correlation which was of marginal significance between MetS and lower CSS in both multivariate Cox regression analysis and Kaplan-Meier survival analysis, indicating that MetS may be associated with increased risk of UTUC.

Evidence based on several current studies showed that patients diagnosed with MetS were more likely to have worse survival outcomes of several types of malignant tumors (10), such as breast cancer (3) and bladder cancer (5). But the inverse relationship between MetS and outcomes were presented in patients with ovarian (11) or renal cancer (12). Even within the same cancer, the results remain controversial. The results of evaluating prognostic value of MetS in localized clear cell renal cell carcinoma (ccRCC) (12, 13) were diametrically opposed. As for UTUC, a recent study based on the Chinese population demonstrated that MetS was a negative prognostic factor of CSS in UTUC and the trend was particularly persisted in patients with non-muscle-invasive UTUC, high-grade disease, and large tumor size (8). As described by another study based on Surveillance, Epidemiology and End Results-Medicare-Linked Database (SEER), MetS and its components were significant risk factors for UTUC among people aged over 65 (7). Whether MetS can be a predictor of UTUC still remains a topic of concern.

MetS, as a cluster of metabolic abnormalities, has complex clinical manifestations and diagnostic criteria. Diagnostic criteria suitable for western populations, which were mostly from large international or European and American institutions, such as the American Heart Association (AHA), the National Heart, Lung, and Blood Institute (NHLBI), and the International Diabetes Federation (IDF). Although some researchers have modified the diagnostic criteria to accommodate local populations, it could also be one of important reasons for differences in results. We adopted the latest version of diagnostic criteria released by CDS, making our best efforts to ensure that the diagnostic criteria was appropriate for the Chinese population. We also collected the use of therapeutic drug for MetS during follow-up according to diagnostic criteria to ensure that MetS could be diagnosed accurately when patients were admitted with normal blood pressure or blood glucose level. MetS may be more easily diagnosed with CDS criteria than others, due to its lower cut-off of BMI. In our study, patients diagnosed with MetS accounted for 32.5% of the total patients, which was higher than 24.4% based on IDF criteria in Xu’s research (8) and 17.1% based on National Cholesterol Education Program-Adult Treatment Panel III (ATPIII) criteria in Lu’s research (7). Difference of diagnostic criteria is expected to affect the level of baseline and clinicopathological characteristics but whether it will result in inconsistency in the prognostic value of MetS on UTUC prognosis still needs further research.

Obesity, as a major component of MetS, has been determined in many studies to be associated with poor prognosis of renal cancer (14) or other cancers. In IDF and ATPIII diagnostic criteria, obesity was defined using waist circumference as the indicator and recent studies have also shown that waist circumference could reflect centripetal obesity more directly than BMI. But the cut-off value of waist circumference was determined from data obtained from the American population. Studies showed that there was significant heterogeneity in waist circumference and BMI among different population and the baseline BMI of the Chinese population was significantly lower than that of western population. A study including 971 Chinese patients showed that BMI was a better predictor of cardiovascular events than waist circumference (9). Unfortunately, the cut-off values of waist circumference or waist-to-hip ratio in Chinese population have not been determined yet, which requires further collaborative research.

A meta-analysis concerning the impact of BMI on urothelial carcinoma provided conclusions that being obese and underweight were predictors for predicted worse survival outcomes, while being overweight was a protective factor (15). But in UTUC patients, previous studies revealed contradictory results. Ehdaie et al. (16) and Dabi et al. (17) showed that increased BMI impacts oncological outcomes in western patients with UTUC, but Kang et al. (18) Liu et al. (19) and Inamoto et al. (20) demonstrated that a preoperative decreased BMI was an independent predictor for OS and CSS after analyzing data from Korean, Chinese, and Japanese populations. Given the large differences in baseline BMI between Asian, and Western populations, collaborative international studies are needed to explained the controversy after rigorous matching. A meta-analysis including 10 studies showed that diabetes increased the risk of IVR in UTUC patients (21), and an international retrospective study discovered that hypertension was a significant risk factor for IVR based on data set from 17 centers worldwide (22). However, the effect of diabetes and hypertension on overall or cancer-specific survival has not been proven. An earlier study showed a weak inverse association between HDL-cholesterol and progress of bladder urothelial carcinoma (23). Xu et al. (8) discovered that patients with hypertriglyceridemia or low HDL-cholesterol were more likely to have adverse pathological features and lower OS, CSS, and RFS of UTUC in univariable Cox regression analyses, but not confirmed in multivariate Cox regression analyses. According to our data and analysis, all components found no statistically significant association with survival outcomes. Further studies are needed to confirm the impact of MetS and its components on progress or prognosis of UTUC.

There is increasing evidence to indicate that there is a certain relationship between MetS and survival prognosis in certain types of cancers. However, numerous mechanisms of how MetS affected pathological features and survival outcomes have been proposed but fail to elaborate at the molecular level, involving insulin-like growth factor (IGF) axis, pro-inflammatory cytokines, circulating factors, angiogenesis, and other important aspects (10). Meanwhile, a network containing these factors and a variety of complex signaling pathways regulates the relation between MetS and tumor progress. The insulin-like growth factor (IGF) system, composed of different subtypes with their receptors and binding proteins, plays an important role on tumor formation, differentiation, and progression. Increased IGF strongly correlates with insulin resistance and obesity, promoting proliferation and migration of pathological cell and overexpression of IGF-1R (24) and IGFBP-5 (24) in UTUC which was proved by *in vitro* experiment. Central fat distribution and low HDL-cholesterol is also associated with production of many pro-inflammatory cytokines, including tumor necrosis factor-α (TNF-α) and interleukin-6 (IL-6), and elevated level of reactive oxygen species (ROS). In addition, MetS patients tend to have high level of adipose tissue, which correlated with an elevated level of serum leptin and reduction of adiponectin. All these factors have been demonstrated to stimulate angiogenesis, which promotes epithelial cell proliferation (10).

Abundant evidence has described the closed association between each single component of MetS with tumor, and combining all the components of MetS as a single condition may not be appropriate. But at the same time, considering MetS and its components as independent factors for UTUC may reveal justification for pre-operative use of MetS-specific drugs including as statin and metformin.

In our study, some potential predicting factors were not included in the multiple Cox analysis, such as N stage, type of bladder cuff management and lymphovascular invasion (LVI). LND is not currently included in UTUC’s standard procedure and is performed only when lymph node metastasis is suspected. Only 89 patients (15.5%) underwent LND as shown in **Tables 1**, **3**, which may result in potential sampling error. Common types of bladder cuff removal included transvesical, extravesical, and endoscopic approaches. The result of a study comparing different methods of bladder cuff management showed that there was no difference in terms of OS, CSS, and RFS among the three distal ureteral management, but patients who underwent the endoscopic approach had a higher risk of IVR (25). Meanwhile, other researchers have also reached different conclusions (26) and the impact of methods of bladder cuff management on oncological outcome is still controversial. The diagnosis of some pathological features including LVI are extremely dependent on pathological reports and sometimes it is difficult to obtain accurate information on some pathological features due to non-standard reporting formats. Smoking, as a factor closely associated with dyslipidemia, is known to increase the risk of experiencing adverse events and IVR in urothelial carcinoma patients (27, 28) but the relation and biological basis between smoking and MetS remains unclear. A recent annual cross-sectional survey showed that life-course cigarette smoking is associated with increased odds of MetS and low high-density lipoprotein-cholesterol (29), which may portend an association between MetS and tumor recurrence.

Our study is not devoid of limitations. First, limited by retrospective data, we could not detail the duration of each component and use of a corresponding drug. Also, small sample size prevented further subgroup analysis based on medication status, such as statin (n=23) or metformin (n=24). Thus, although we adjusted preoperative confounders by propensity score matching with a small caliber of 0.02, some confounders which may affect survival outcomes, like tobacco consumption and some pathological features, were not included. Finally, the duration, dose, and regimen of adjuvant chemotherapy were incomplete, so we were not able to evaluate its potential mitigation effect on survival outcomes.

## Conclusion

In our study, no statistical difference was found between MetS and survival outcomes in UTUC, except a marginal association with lower CSS. Further studies are needed to evaluate the role of MetS and its each single components on UTUC.

## Data Availability Statement

The raw data supporting the conclusions of this article will be made available by the authors, without undue reservation.

## Ethics Statement

The studies involving human participants were reviewed and approved by Ethics committee of Peking University People’s Hospital. Written informed consent for participation was not required for this study in accordance with the national legislation and the institutional requirements.

## Author Contributions

XD carried out the study, participated in the data analysis and drafted the manuscript. XD, YD, FW, SH, and ZH collected the data. XD, YD, CQ, SL, YS, and TX participated in designing the study. XD, YD, XZ, and TX revised the manuscript for important intellectual content. All authors gave final approval of the version to be published, and agree to be accountable for all aspects of the work.

## Funding

This study was funded by the National Key Research and Development Program of China (No. 2018YFA0902802), Natural science foundation of Beijing, China (No. 7202219), National natural science foundation of China (No. 81802533), and Beijing Municipal Science & Technology Commission (No. Z191100006619010).

## Conflict of Interest

The authors declare that the research was conducted in the absence of any commercial or financial relationships that could be construed as a potential conflict of interest.

## Publisher’s Note

All claims expressed in this article are solely those of the authors and do not necessarily represent those of their affiliated organizations, or those of the publisher, the editors and the reviewers. Any product that may be evaluated in this article, or claim that may be made by its manufacturer, is not guaranteed or endorsed by the publisher.
